# Ketoconazole Reverses Imatinib Resistance in Human Chronic Myelogenous Leukemia K562 Cells

**DOI:** 10.3390/ijms23147715

**Published:** 2022-07-13

**Authors:** Omar Prado-Carrillo, Abner Arenas-Ramírez, Monserrat Llaguno-Munive, Rafael Jurado, Jazmin Pérez-Rojas, Eduardo Cervera-Ceballos, Patricia Garcia-Lopez

**Affiliations:** 1 Laboratorio de Fármaco-Oncología, Subdirección de Investigación Básica, Instituto Nacional de Cancerología, Ciudad de Mexico 14080, Mexico; oprado@live.com.mx (O.P.-C.); alex_abner.ar@ciencias.unam.mx (A.A.-R.); muniv1250@hotmail.com (M.L.-M.); fcojl@yahoo.com (R.J.); jazminmarlen@gmail.com (J.P.-R.); 2 Posgrado en Ciencias Bioquímicas, Universidad Nacional Autónoma de México (UNAM), Ciudad de Mexico 04510, Mexico; 3 Dirección de Docencia, Instituto Nacional de Cancerología, Ciudad de Mexico 14080, Mexico; eduardocer@yahoo.com

**Keywords:** chronic myeloid leukemia, imatinib, tyrosine kinase, ketoconazole, P-glycoprotein, drug efflux transporter

## Abstract

Chronic myeloid leukemia (CML) is a hematologic disorder characterized by the oncogene BCR-ABL1, which encodes an oncoprotein with tyrosine kinase activity. Imatinib, a BCR-ABL1 tyrosine kinase inhibitor, performs exceptionally well with minimal toxicity in CML chemotherapy. According to clinical trials, however, 20–30% of CML patients develop resistance to imatinib. Although the best studied resistance mechanisms are BCR-ABL1-dependent, P-glycoprotein (P-gp, a drug efflux transporter) may also contribute significantly. This study aimed to establish an imatinib-resistant human CML cell line, evaluate the role of P-gp in drug resistance, and assess the capacity of ketoconazole to reverse resistance by inhibiting P-gp. The following parameters were determined in both cell lines: cell viability (as the IC50) after exposure to imatinib and imatinib + ketoconazole, P-gp expression (by Western blot and immunofluorescence), the intracellular accumulation of a P-gp substrate (doxorubicin) by flow cytometry, and the percentage of apoptosis (by the Annexin method). In the highly resistant CML cell line obtained, P-gp was overexpressed, and the level of intracellular doxorubicin was low, representing high P-gp activity. Imatinib plus a non-toxic concentration of ketoconazole (10 μM) overcame drug resistance, inhibited P-gp overexpression and its efflux function, increased the intracellular accumulation of doxorubicin, and favored greater apoptosis of CML cells. P-gp contributes substantially to imatinib resistance in CML cells. Ketoconazole reversed CML cell resistance to imatinib by targeting P-gp-related pathways. The repurposing of ketoconazole for CML treatment will likely help patients resistant to imatinib.

## 1. Introduction

Chronic myeloid leukemia (CML), also known as chronic granulocytic leukemia, is a myeloproliferative disorder. It is characterized by neoplastic growth of myeloid cells in the bone marrow, leading to a significant increase of these cells in peripheral blood [1]. CML is traditionally described as a triphasic disease, beginning with the chronic phase and progressing to the accelerated phase, and finally to the blast phase. Chemotherapy given at the early chronic phase usually restores the patient to a normal-like state, which can be sustained for months or years. Nevertheless, without medical treatment or a lack of response to treatment, patients gradually progress to blast crisis [1,2].

The disease has its origins in the formation of the BCR-ABL1 gene, which results from the reciprocal translocation between chromosomes 9 and 22 and the fusion of the ABL1 and BCR genes to create a short chromosome called Philadelphia chromosome. The fused BCR-ABL1 gene codes for an abnormal oncoprotein with tyrosine kinase activity (Bcr-Abl) and with auto-phosphorylation capacity. Bcr-Abl activates multiple signaling pathways that cause the abnormal proliferation of hematopoietic stem cells (HSC) and thus the manifestation of the disease [3,4]. The BCR-ABL gene is present in all cases of CML. It provides a unique biomarker for diagnosis and is targeted during treatment with tyrosine kinase inhibitors (TKIs) to selectively inhibit Bcr-Abl. The first TKI, imatinib mesylate, became the basis of therapy for CML, transforming this disease from a fatal to a chronic one [5].

In 1996, Druker et al. [6] reported the in vitro effects of a specific inhibitor of the BCR-ABL tyrosine kinase on CML cell lines for the first time. This inhibitor was known as signal transduction inhibitor (STI571) but is now called imatinib (Gleevec^®^). In a phase 1 study of the advanced stage of the disease, STI571 not only controlled blood counts and restored the chronic phase, but 95% of patients achieved a complete hematologic response and a 60% greater cytogenetic response. Despite the short follow-up period existing at that time, imatinib was granted accelerated approval by the FDA in 2001 due to its exceptional efficacy and minimal toxicity [7]. It was established as the first-line treatment for CML [8,9].

The drug occupies the ATP-binding site on the BCR-ABL protein. The resulting conformational change in the tyrosine kinase quaternary structure inhibits autophosphorylation and phosphorylation of tyrosine residues on protein substrates. Thus, imatinib prevents the transduction of signals crucial for the abnormal and uncontrolled cell proliferation caused by the BCR-ABL gene in CML cells [10].

Since the bioavailability of imatinib is around 92% (86–99%) with a half-life of 18 h in healthy volunteers and patients [11], one dose/day seems to be appropriate. The drug is extensively metabolized by cytochrome P450 enzymes (CYP-P450). Additionally, it is a substrate of the drug transporter denominated P-glycoprotein (P-gp or MDR1 [multidrug resistance 1]), an ATP-dependent efflux pump that decreases intracellular drug concentrations [12]. Hence, P-gp influences drug absorption, distribution, metabolism, and excretion (ADME). Even with the high bioavailability of imatinib, its pharmacokinetics show a great variability in the responses of individuals, which are affected by both CYP-P450 and the P-gp efflux transporter [13].

The surprising efficacy of imatinib in CML is attenuated by resistance in a percentage of patients with advanced-stage CML [14,15,16]. The known mechanisms of resistance to imatinib can be divided into those BCR-ABL-dependent and BCR-ABL-independent. BCR-ABL-dependent mechanisms include mutations in the ABL kinase and/or mutations and amplification of the BCR-ABL gene. Among BCR-ABL-independent mechanisms is drug efflux mediated by ATP-binding cassette (ABC) transporters [17].

Specifically, it has been reported that the P-gp protein (encoded by the ABCB1 gene) may contribute considerably to resistance to imatinib. P-gp is capable of diminishing the intracellular concentration of imatinib by pumping it out of leukemia cells [17,18]. This efflux pump is located in normal human tissue in the liver, kidney, colon, adrenal gland, intestine, placenta, endothelial cells of the blood-brain barrier, and hematopoietic precursor cells. However, its expression is significantly elevated in drug-resistant tumors, making it an obstacle to successful chemotherapy. Therefore, a reduction of the level of P-gp by inhibitors could lead to the sensitization of CML resistant cells to imatinib and therefore the avoidance of resistance.

Numerous studies have identified various competitive substrates and inhibitors of P-gp, allowing for a greater understanding of the regulation of P-gp functions to overcome drug resistance. Several of these compounds are drugs originally approved for clinical indications unrelated to cancer [19], as is the case with ketoconazole. In 2004, Dutreix et al. described an increase in the plasma concentration of imatinib in healthy volunteers who had taken ketoconazole. The mechanisms involved were the blocking of imatinib metabolism by CYP3A4 and the inhibition of its extrusion from gastrointestinal cells by impeding P-gp activity [20]. To date, however, there have been no reports on the capacity of ketoconazole to reverse the resistance of CML cells to imatinib treatment. Thus, the purpose of the current study was first to generate an in vitro model of CML cells resistant to imatinib, and secondly to explore the participation of the drug efflux transporter P-gp in such resistance. Subsequently, an evaluation was made of the capacity of ketoconazole to overcome resistance to imatinib through the inhibition of P-gp. Finally, the possible role of ketoconazole in triggering the apoptosis of imatinib-resistant cells was assessed.

## 2. Results

### 2.1. The Development of CML K562 Cell Resistance to Imatinib

By treating the K562 parent cell line with gradually increasing concentrations of imatinib (ranging from 1 to 2500 nM), the resistant phenotype was developed in about 8 months. Drug resistance was confirmed by a cell viability assay based on a 72 h exposure of sensitive (K562) and resistant (K562-RI) cells to imatinib. A clear difference between the two cell viability curves was observed, with the graph displaying a lesser effect of imatinib on the K562-RI cell line.

**Figure 1:**
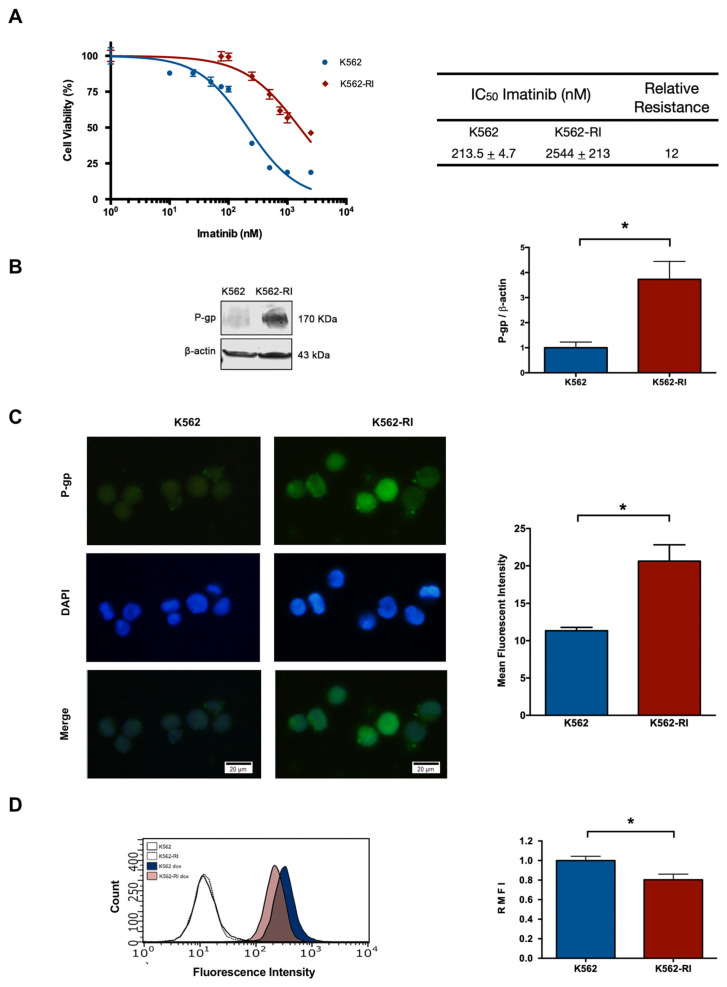
**The development of a CML cell line with resistance to imatinib treatment.** (**A**) Percentage of cell viability after applying imatinib alone (at distinct concentrations), considering CML cells sensitive (K562) and resistant (K562-RI) to this drug. The table denotes the IC50 of imatinib in each cell line and the relative resistance of K562-RI (calculated as the IC50 of K562-RI divided by that of K562). (**B**) P-gp expression levels in the K562 and K562-RI cell lines, based on Western blot and its densitometric analysis (β-actin was the load control); three Western blots from three independent experiments were used for densitometric analysis. (**C**) Analysis of P-gp evaluated by the immunofluorescence assay (the nuclei were visualized with DAPI). The mean fluorescence intensity was quantified by counting P-gp-positive cells from three independent experiment. Scale bars: 20 μm. (**D**) Representative histograms of the fluorescence of uptake of doxorubicin in the K562 and K562-RI cell lines. The empty histograms depict the control cells without doxorubicin. Data are expressed as the relative mean fluorescence intensity (RMFI) ± SEM of three independent experiments. The values of k562-RI were normalized with respect to K562. Statistical analysis was performed by comparing K562-RI to the parental cell line. * *p* < 0.05; Student’s *t*-test.

The IC50 of imatinib was determined for the K562-RI and K562 cell lines, finding the values of 2544 nM and 213 nM, respectively. Hence, there was an approximately 12-fold relative resistance, calculated as the ratio of the IC50 values of resistant and sensitive cells (Figure 1A).

### 2.2. The Expression of P-Glycoprotein in Resistant and Susceptible CML Cells

After confirming the resistant phenotype, the expression of P-gp was examined by Western blot, finding it to be around 4-fold greater in K562-RI than K562 cells (Figure 1B), correlating with the higher level of P-gp in resistant cells observed by immunofluorescence (Figure 1C). Also indicating a higher level of P-gp in resistant cells was the test with doxorubicin, which showed a lower intracellular accumulation of this compound in resistant than susceptible CML cells, as can be appreciated by the values of relative mean fluorescence intensity (RMFI) (Figure 1D).

### 2.3. Effect of Ketoconazole on K562 Cells (Sensitive to Imatinib)

**Figure 2:**
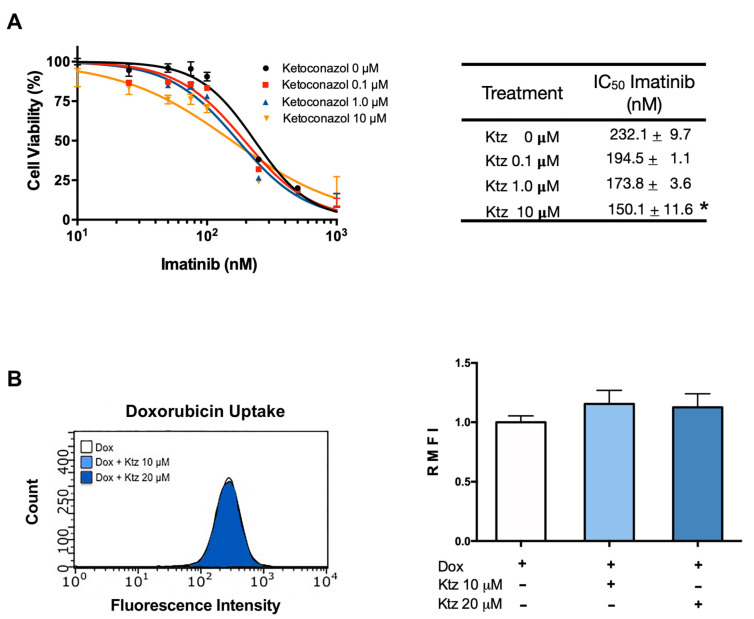
**Effect of ketoconazole on K562 cells (sensitive to imatinib)**. (**A**) Cell viability of the K562 cell line exposed to imatinib in the absence or presence of ketoconazole (at 0.1, 1.0, and 10 μM). The table shows the IC50 of imatinib alone and imatinib plus ketoconazole. (**B**) Effect of ketoconazole (at 10 and 20 μM) on the fluorescence of doxorubicin in K562 cells, representative histograms with raw data and in a bar graph. Data are expressed as the relative mean fluorescence intensity (RMFI) ± SEM of three independent experiments. The cells treated with doxorubicin plus ketoconazole 10 and 20 µM were normalized against doxorubicin alone. Ktz, ketoconazole; Dox, doxorubicin. * Significant difference (*p* < 0.05) by one-way analysis of variance (ANOVA) followed by Tukey’s test.

The application of imatinib brought about a dose-dependent antiproliferative effect in K562 cells (Figure 2A), which was unmodified by the combination treatment with ketoconazole at 0.1 and 1.0 μM (Figure 2). However, significantly increased antiproliferative activity was detected with imatinib plus ketoconazole at 10 μM (Figure 2A). In this cell line, the IC50 of imatinib monotherapy was 232 nM, while that of imatinib co-incubated with ketoconazole at 10 μM was 150 nM, translating into a 0.65-fold reduction. According to the quantification of intracellular doxorubicin in sensitive cells, no change took place with ketoconazole at 10 or 20 μM (Figure 2B).

### 2.4. Ketoconazole Induced a Reversal of the Resistance of K562-RI Cells to Imatinib

**Figure 3:**
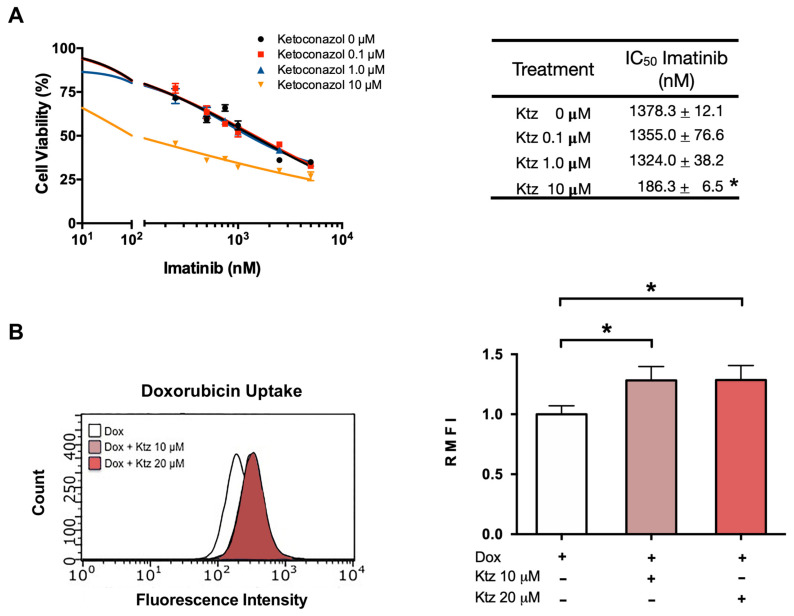
**Ketoconazole-induced reversal of K562-RI cell resistance to imatinib**. (**A**) Cell viability of the resistant cell line (K562-RI) exposed to imatinib in the absence and presence of ketoconazole (at 0.1, 1.0 and 10 μM). The table shows the IC50 of imatinib alone and imatinib plus ketoconazole. (**B**) Effect of ketoconazole on the fluorescence of doxorubicin in K562-RI cells. The bar graph portrays the relative fluorescence of doxorubicin in the presence of 10 or 20 μM of ketoconazole. Data are expressed as the relative mean fluorescence intensity (RMFI) ± SEM of three independent experiments. The cells treated with doxorubicin plus ketoconazole 10 and 20 µM were normalized against doxorubicin alone. Statistical analysis was performed by comparing the level of intracellular doxorubicin between the treatment with doxorubicin plus ketoconazole (10 or 20 μM) and doxorubicin alone. * *p* < 0.05; determined with ANOVA followed by Tukey’s test. Im, imatinib; Ktz, ketoconazole; Dox, doxorubicin.

As with K562 cells, the antiproliferative effect of imatinib on K562-RI cells was not improved by co-treatment with ketoconazole at 0.1 or 1.0 μM. However, a reversal of drug resistance in K562-RI cells was produced by applying imatinib plus ketoconazole at 10 μM (Figure 3A), resulting in an IC50 value of 186 nM. Considering the IC50 value of 1378 nM found after applying imatinib alone to K562-RI cells, the combination treatment afforded an approximately 7.5-fold reversal index.

### 2.5. Effect of Ketoconazole on P-Glycoprotein Expression in Resistant Cells

**Figure 4:**
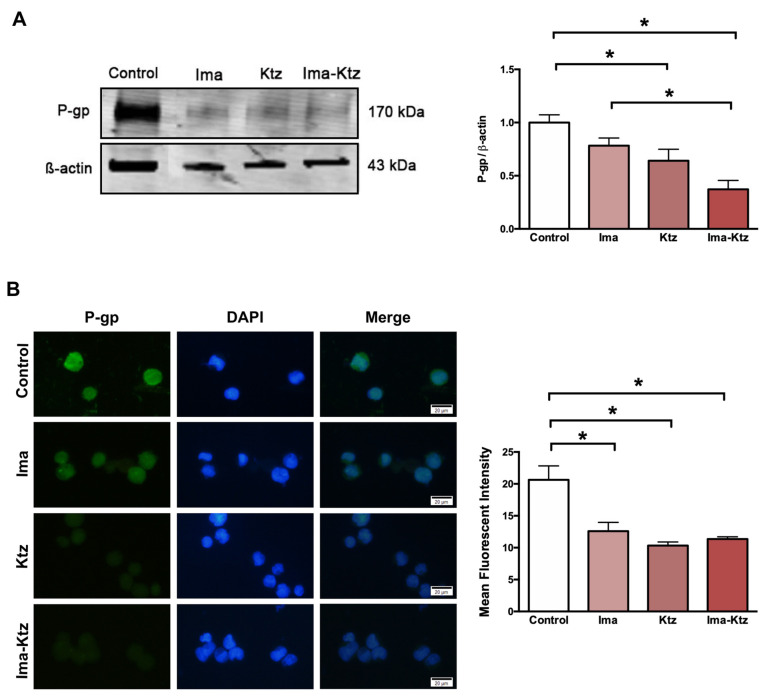
**P-gp expression in resistant cells treated with imatinib and ketoconazole.** (**A**) P-gp expression levels in K562-RI cells exposed to imatinib in the absence and presence of ketoconazole based on Western blot and its densitometric analysis (β-actin was the load control); three Western blots from three independent experiments were used for densitometric analysis ± SEM. (**B**) analysis of P-gp evaluated by the immunofluorescence assay (DAPI was used to visualize the nuclei). The Mean Fluorescent Intensity was quantified by counting P-gp-positive cells (in 40–45 cells per group counted randomly) from three independent experiments. The cells K562-RI cells were exposed to imatinib alone (200 nM), ketoconazole alone (10 μM), or imatinib plus ketoconazole (200 nM/10 μM). * *p* < 0.05; determined with ANOVA followed by Tukey’s test. Scale bars: 20 μm. Ima, imatinib; Ktz, ketoconazole.

The Western blot (Figure 4A) and immunofluorescence assay (Figure 4B) performed with K562-RI cells demonstrated that imatinib alone (at 200 nM) and ketoconazole alone at 10 μM slightly reduced the expression of P-gp, while significantly diminished the level of P-gp, with imatinib plus ketoconazole causing a greater decrease (Figure 4).

### 2.6. Effect of Ketoconazole on Apoptosis in Resistant Cells

**Figure 5:**
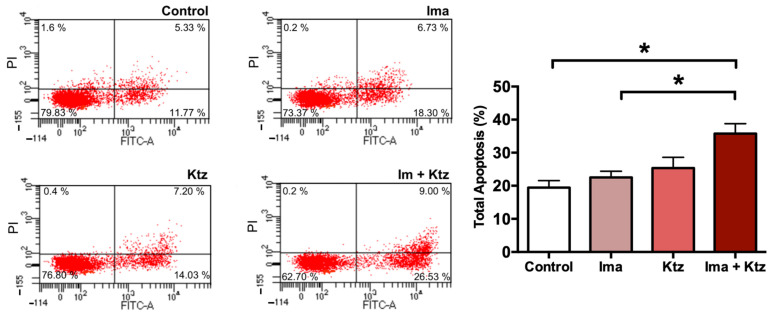
**Flow cytometric analysis of apoptosis in K562-RI cells at 96 h post-treatment.** Representative histograms depicting cells positive to Annexin V-FITC/IP (early and late apoptosis) by flow cytometric analysis; and percentage of total apoptosis. * *p* < 0.05, examined with one-way analysis of variance (ANOVA) followed by Tukey’s test. Data are expressed as the mean ± SEM of three independent experiments. Ima, imatinib (200 nM); Ktz, ketoconazole (10 μM); Ima + Ktz, the combination of imatinib plus ketoconazole (200 nM/10 μM).

Since imatinib plus ketoconazole at 10 μM significantly inhibited the viability of K562-RI cells, apoptosis was explored as a possible mechanism of action. Externalization of phosphatidylserine to the outer surface of the plasma membrane is a clear sign of early apoptosis. K562-RI cells were stained with Annexin V-FITC (early apoptosis) and PI (late apoptosis) for flow cytometric analysis, which revealed the lack of any significant change in the percentage of apoptosis produced by imatinib alone or ketoconazole alone (versus the resistant control cells exposed to the vehicle only). However, there was indeed a significantly greater programmed cell death of resistant cells when using imatinib plus ketoconazole (~40%). These data suggest that exposure of cells to ketoconazole triggered apoptosis (Figure 5).

## 3. Discussion

Before 2001, the median survival of patients with CML was 5–7 years. Due to the introduction of TKI therapy, overall survival 5 years after treatment is now 92–95% (1). To date, the Food & Drug Administration (FDA) has approved four TKIs as first-line drugs for CML: imatinib, dasatinib, nilotinib and bosutinib. Imatinib is the first-generation drug, being the first to be approved in 2001 [21]. Whereas annual mortality for CML patients was 10–20% prior to the introduction of imatinib, it is now 1–2% [22].

There are several aspects to be considered in selecting one of the four inhibitors for CML therapy. Imatinib was the first TKI to receive approval by FDA for the treatment of patients with CML in chronic phase, is recommended for older patients with comorbidities, being the safest drug with the fewest side effects [23]. The use of dasatinib or nilotinib, TKI of the second generation, is justified in high-risk patients or young patients in need of a deep response with a first-line treatment. Whereas bosutinib, a third generation TKI, is intended for patients with chronic-, accelerated-, or blast-phase CML who cannot tolerate or are resistant to other therapies [24,25].

Most patients administered imatinib can carry on normal lives as long as they adhere to treatment. Since the dosing is carried out indefinitely, however, adherence is complicated, especially if patients experience side effects. In the latter case, adherence is likely to decline over time, leading a percentage of patients to develop resistance. The ADAGIO study showed that only 14.2% of patients took 100% of the prescribed imatinib doses. Furthermore, non-adherent patients constituted 23.2% of those with a suboptimal response and only 7.3% of those with an optimal response [26]. According to another study conducted on CML patients administered imatinib for a few years, poor adherence may be the predominant reason for a low level of response to imatinib and the development of resistance to the same [27].

Based on clinical trials, around 20–30% of patients eventually develop drug resistance [28], which consists of an absence of the initial desired response to the drug in a certain period of time (primary resistance), or the presence of the desired response followed by its loss (secondary resistance). Among the patients with primary or secondary resistance, many progress to accelerated phase and blast phase [15].

Researchers seeking new strategies to confront the emergence of resistance to imatinib have been obliged to gain new insights into the molecular mechanisms of resistance. Although there are several reports of resistance to imatinib being dependent on BCR-ABL1, the overexpression of P-gp (a drug efflux pump) could also be a crucial resistance mechanism [29,30,31], because it regulates the intracellular concentration of the drug.

In the current contribution, the first step was to establish drug resistance in the K562 cell line through its constant exposure to imatinib (for about eight months). The resistant cell line exhibited a drastic drop in its response to imatinib, evidenced by a significant increase in the IC_50_ from 213 nM (K562) to 2544 nM. As can be appreciated, an approximately 12-fold greater dose is necessary to attain the same result with the K562-RI cells compared to the K562 cells.

The next step was to examine differences between the two cell lines in relation to certain parameters. In a recent clinical study on CML patients by Ammar et al. (2020), an association was found between elevated levels of P-gp and unresponsiveness to treatment, indicating that the overexpression of P-gp is probably a relevant mechanism in the development of resistance to imatinib [32]. According to the present data from Western blot analysis and immunofluorescence assays, P-gp levels were almost undetectable in the parent cells but were about 4-fold higher in K562-RI cells.

A high level of P-gp implies that its substrates can easily be removed from cells. Like imatinib, doxorubicin is a P-gp substrate and is efficiently pumped out of tumor cells when P-gp is highly expressed, leading to a reduced intracellular accumulation. To evaluate the activity of P-gp, the intracellular accumulation doxorubicin was herein analyzed after incubation of cells with this compound, finding a lower mean fluorescence intensity of intracellular doxorubicin in K562-RI versus K562 cells (Figure 1D). This low uptake of doxorubicin was accompanied by an elevated level of P-gp. Similar results have been reported by other researchers using in vitro models [30,31]. Moreover, K562-RI cells were incubated in doxorubicin only and doxorubicin plus ketoconazole, finding a greater intracellular fluorescence in the latter group with the P-gp inhibitor. Hence, an overexpression of the P-gp drug efflux pump appears to be an important mechanism of resistance of CML cells to imatinib.

The next question to be explored was the effect of applying a P-gp inhibitor to the K562 and K562-RI cell lines. P-gp, the best characterized molecule of the class of efflux pump transporters, is known to produce resistance to treatment by removing drugs from various kinds of cancer cells resistant to drug treatment. Consequently, several studies have evaluated the inhibition of this transporter to try to enhance the anti-proliferative activity of chemotherapy for distinct types of cancer. Some of these studies have tested compounds possibly capable of improving the pharmacokinetics of ITKs. However, there are few reports on the inhibition of P-gp to overcome the resistance of CML cells to imatinib.

Several researchers have demonstrated that ketoconazole is a strong inhibitor not only of CYP-P450 3A4 (CYP3A4) but also of P-gp [33]. On the other hand, imatinib is a substrate of P-gp and is metabolized by CYP-P450, mainly by the CYP3A4 isoenzyme. Thus, imatinib should be susceptible to drug interactions if administered concomitantly with potent inhibitors or inducers of CYP-P450 and/or P-gp. In this sense, the concomitant intake of imatinib and ketoconazole was found to increase the plasma concentration of imatinib in healthy subjects [20].

In the current contribution, imatinib plus 10 μM of ketoconazole was applied to resistant (K562-RI) CML cells with the aim of decreasing the overexpression of P-gp, enhancing the intracellular concentrations of imatinib, and accelerating the rate of programmed cell death. The result was the reversal of resistance to the standard drug. The IC50 of imatinib dropped from 1378 nM (without ketoconazole) to 186 nM (with ketoconazole), reflecting an approximately 7.5-fold reversal index. In K562 cells (with negligible levels of P-gp), this combination regimen only slightly improved the effect of imatinib. Contrarily, K562-RI cells subjected to the combination treatment showed a clear reduction in the expression of P-gp in Western blot and immunofluorescence assays (Figure 4), and a significantly greater relative fluorescence of intracellular doxorubicin after applying ketoconazole (10 and 20 μM) plus doxorubicin (versus doxorubicin alone) (Figure 3). Similarly, Siegsmund et al. found that applying ketoconazole in combination with vinblastine or doxorubicin to treat a highly resistant human cell line (KB-Vl) enhanced the intracellular accumulation of the latter two cytotoxic drugs. Both combinations reversed multidrug resistance with ketoconazole at 1, 3, and 10 µg/mL (∼2, 6, and 19 µM, respectively) [34], concentrations comparable to those used in the present work. In another study, it was also shown that ketoconazole was able to inhibit P-gp at a concentration of 6 µM in NIH-3T3-G185 cells that overexpressing human P-gp [35]. It has also been described that ketoconazole at 10 and 20 µM strongly enhanced cell growth inhibition and apoptosis of paclitaxel or cisplatin in ovarian cancer cells through its pregnane X receptor (PXR) antagonism. This nuclear receptor affects drug metabolism/efflux and drug-drug interaction through P-gp expression [36]. The lowest concentration of ketoconazole that presently demonstrated a positive effect was 10 µM, which is within the range of human plasma concentrations observed after a single oral dose of 200–400 mg [37]. Therefore, these non-toxic concentrations of ketoconazole can easily be achieved with clinical pharmacological doses of the drug and may be clinically well-tolerated if administered in combination with imatinib to treat patients with resistance to the standard drug.

Some additional mechanism by which ketoconazole is able to reverse resistance of imatinib could be related to regulation of P-gp/CYP3A4 by PXR, which regulates the expression of metabolic enzymes and transporters [38]. Ketoconazole has been reported as an inhibitor of PXR [39,40]. Therefore, it could regulate the transcription of the P-gp/CYP3A4 gene through disruption of PXR interaction with steroid receptor coactivator (SRC)-1.

A fundamental characteristic of cancer cells is their capacity to avoid apoptotic cell death. The cooperation between P-gp and molecules capable of inhibiting apoptosis-related proteins can generate a more robust drug resistance in cancer cells in general, and CML cells in particular. Among the numerous mechanisms utilized by CML cells to become resistant to imatinib, the avoidance of apoptosis is probably one of the most common.

The present study explored apoptosis as a possible mechanism of growth inhibition by the combination treatment. The Annexin V/PI assay, used for assessing early and late apoptosis, showed that imatinib plus ketoconazole increased apoptosis by 40%, whereas treatment with only imatinib or ketoconazole produced 25% greater apoptosis compared to control cells (Figure 5). Consequently, a plausible mechanism of ketoconazole for improving the efficacy of imatinib is its capacity to trigger apoptosis. Previous studies have reported that ketoconazole produce apoptosis inducing p53 levels and PARP cleavage in breast cancer cells [41], human colorectal and hepatocellular carcinoma cell lines [42]; therefore, a similar mechanism could be involved in the apoptosis of CML

On the other hand, Chen et al. recently reported that ketoconazole accelerates the process of apoptosis in hepatocarcinoma cells by exacerbating mitophagy, activating the PINK1/Parkin (PRKN) signaling pathway and downregulating cyclooxygenase-2 (COX-2), an inducible form of the enzyme that catalyzes the synthesis of prostanoids. The overexpression of COX-2 has been related with resistance to apoptosis [43]. In addition to these results, several reports reveled that the overexpression of COX-2 leads to increased P-gp expression [44]. Likewise, an overexpression of COX-2 and P-gp has been demonstrated in resistant K562 cells, with a decrease in apoptosis involving the Akt/p-Akt signaling pathway, which suggests the participation of COX-2 and P-gp in the development of resistance of CML cells [45]. We propose that ketoconazole stimulates apoptosis through COX-2 inhibition, in addition to P-gp inhibition, in imatinib-resistant cells (K562-RI).

Ketoconazole is an antifungal drug known to impede fungal growth by preventing the synthesis of ergosterol (the fungal equivalent of cholesterol) [46,47]. According to recent reports and the current findings, this drug seems to have great potential for cancer therapy [48,49,50,51,52,53].

Since its approval by the FDA in 1982, the estimated number of prescriptions of ketoconazole in the United States has been increasing every year. The oral use of ketoconazole is well tolerated in patients with limited toxicity. In the CALGB 9583 trial, 2% of patients who received 400 mg of ketoconazole three times per day, had grade 3 or 4 hepatotoxicity. However, the low ketoconazole dose (200 mg) was less toxic; thus, for patients who cannot tolerate high dose treatment, the low dose would be an option [54]. In another report, Outeiro et al. reported that only 1.7% of patients who received ketoconazole (400 mg/day for 28 days) experienced liver function abnormalities [55]. Several reports in the literature have also described that ketoconazole has mild toxicity and is rarely fatal in comparison with other azoles as voriconazole, fluconazole, itraconazole among others. Ketoconazole toxicity can be reversed upon drug discontinuation [56,57,58].

Thus, ketoconazole is a safe drug with a relatively low cost compared to the high price of new medications for cancer. The price of new anti-cancer drugs reflects the corresponding research and development costs, generally around a billion dollars or more [59,60]. One strategy now employed more and more frequently is the repositioning of drugs, which involves giving approved drugs new applications. Among the advantages is the known profile of safety and efficiency.

The literature describes a large number of P-gp substrates already approved by the FDA; nevertheless, to date there are few reported studies evaluating P-gp inhibition in CML patients. Cyclosporine was one of the first drugs studied concerning clinical P-gp modulations; despite the results in cells, it also showed high toxicity [61,62]. Recently several in vitro studies, using P-gp inhibitors, supported the role of efflux activity of this protein in CML resistance. Liu et. al, in 2018, demonstrated that non-toxic concentrations of nelfinavir, an anti-HIV drug, reverse the resistance of adriamycin (doxorubicin), colchicine, paclitaxel and imatinib in k562/ADR cells that overexpressed P-gp. Nelfinavir, in addition to being a protease inhibitor drug approved for the treatment of AIDS patients, it has also been proposed as a new antitumor drug for the treatment of CML [63]. Elacridar, a potent P-gp inhibitor, approved recently by FDA, has demonstrated interesting results about to promote a synergic effect with imatinib in resistant cells [64]. Azithromycin, antimicrobial drug, may be another interesting alternative to overcome imatinib resistance according to the results described of its ability to inhibit P-gp function and increase intracellular accumulation of imatinib [65]. Although all these drugs (including ketoconazole), show interesting results as potential drugs for the treatment of resistant CML, clinical trials are needed to demonstrate their effectiveness in reversing resistance by targeting P-gp and prove their low systemic toxicity in CML patients.

## 4. Materials and Methods

### 4.1. Cell Lines

Human CML K562 cells were acquired from the American Type Culture Collection (ATTC, Rockville, MD, USA) and they were cultured in Dulbecco’s modified Eagle’s medium (DMEM) supplemented with 10% fetal bovine serum, at 37 °C in a humidified atmosphere with 5% CO_2_.

### 4.2. Development of the CML Cell Line with Resistance to Imatinib Treatment

The K562 cells were seeded in a culture flask with a surface area of 25 cm^2^, they were exposed to gradually increasing concentrations (ranging from 1 nM to 2500 nM) of imatinib over various months, establishing an imatinib-resistant culture. After about eight months, the resistant phenotype, designated as K562-RI, was confirmed with the cell viability assay. To maintain the resistance of the K562-RI cells, 250 nM of imatinib were added to the culture medium.

To evaluate the resistance of K562-RI to imatinib, these cells were seeded into 96-well plates (Costar, Cambridge, MA, USA) at a density of 12 × 10^3^ viable cells per well in 150 μL of culture medium. They were exposed to increasing concentrations of imatinib for 72 h. Cell viability to test cell resistance to imatinib was assessed with the sodium 3′-[1-[(phenylamino)-carbony]-3,4-tetrazolium]-bis(4-methoxy-6-nitro) benzene-sulfonic acid hydrate (XTT) assay (Roche Molecular Biochemicals), which is based on the cleavage of yellow tetrazolium salt XTT to form an orange formazan dye by metabolically active cells.

Briefly, 50 μL of XTT were added to each well with K562-RI cells (for a final concentration 0.3 mg/mL), followed by incubation at 37 °C for 2 h in a humidified atmosphere containing 5% CO_2_. The parent K562 cell line was also tested with the XTT assay for comparison. After quantifying the absorbance of the samples from both cell lines spectrophotometrically at 492 nm with an ELISA microplate reader (Thermo Scientific, Waltham, MA, USA), the percentage of viability and relative resistance was calculated. Data are expressed as the mean ± SEM of three independent experiments performed in triplicate. For each experiment, the resistance of the K562-RI cell line was confirmed using the XTT assay to calculate cell viability and corroborate relative resistance.

Additionally, the level of P-gp was determined by Western blot; three independent experiments were performed, and the intracellular accumulation of doxorubicin was evaluated as an indirect measure of P-gp activity (given that doxorubicin is considered a substrate for P-gp transport). Data are expressed as the mean ± SEM of three independent experiments.

### 4.3. Treatments with Imatinib and Ketoconazole

K562 and K562-RI cells were seeded into 96-well plates (Costar, Cambridge, MA, USA) at a density of 12 × 10^3^ viable cells per well in 150 μL culture medium, and then exposed for 72 h to various amounts of imatinib alone (0–2500 nM) or imatinib plus ketoconazole (0.1, 1.0, and 10 μM). Control cells were only in contact with the vehicle. At the end of the exposure period, cell viability was examined with the XTT assay, as aforementioned. The mean concentration in each set of four wells was determined in triplicate. The dose–response relationship for imatinib applied alone or in combination with ketoconazole was characterized with a sigmoidal function. The percentage of growth inhibition was calculated, and the IC50 values (the concentration of the drug required to afford 50% growth inhibition) were obtained from the survival curve fitted to a non-linear regression using the GraphPad Software, Prism 7.0 (San Diego, CA, USA) The equations used were: “Dose-response curves-Inhibition” and “log(inhibitor) vs. normalized response”; with the following function: Y = 100/(1 + 10^((LogIC50-X) × HillSlope))). Where the HillSlope describes the steepness of curves, and the IC50 is the concentration that provokes a response halfway between the minimum and maximum response [66].

### 4.4. P-Glycoprotein Expression Analyzed by Western Blotting

To evaluate the P-gp expression levels in resistant cells, K562 and K562-RI cells (1 × 10^6^ cells) were seeded and incubated for 24 h, they were recollected and centrifuged. The resulting pellets were washed three times with PBS, then homogenized with a lysis buffer containing protease inhibitors. To evaluate the P-gp expression levels in K562-RI cells exposed to imatinib in the absence and presence of ketoconazole, K562-RI cells (1 × 10^6^ cells) were cultured overnight, then treated with imatinib (200 nM), ketoconazole (10 μM), or imatinib plus ketoconazole (200 nM/10 μM) during 4 h, then the cells were recollected, washed with PBS and homogenized with a lysis buffer. The proteins were separated by centrifugation at 10,000× *g* and 4 °C, quantified by the BCA (bicinchoninic acid) assay, and separated electrophoretically on 4–20% gradient gel (Mini-Protean TGX 456-1094, Bio-Rad Laboratories, Inc., Burns, TN, USA). Markers (Bio-Rad, Hercules, CA, USA) were included to establish protein size. Subsequently, the proteins were transferred from the gel onto PVDF membranes (Amersham, UK), which were blocked with 5% non-fat dry milk at room temperature for 2 h. Membranes were incubated overnight at 4 °C with antibodies against P-gp (12683, 1:500, Cell Signaling Technology, Danvers, MA, USA) and β-actin (sc-69879, 1:1000; Santa Cruz Biotechnology, Dallas, TX, USA). The membranes were washed and incubated with IRDye^®^ 800 CW goat anti-mouse or IRDye^®^ 680RD goat anti-rabbit secondary antibodies (1:15,000; LI-COR, Biotechnology, Inc., Lincoln, NE, USA) for 1 h and then scanned on an Odyssey Imaging System. Their intensity of fluorescence was calculated by using Image Studio software. In each figure, representative blot images were selected from the same gel.

### 4.5. P-Glycoprotein by Immunofluorescence Assay

K562-RI cells (1 × 10^6^ cells) were cultured overnight in culture slides (CultureSlides, Falcon Corning, NY, USA), then treated with imatinib (200 nM), ketoconazole (10 μM), or imatinib plus ketoconazole during 4 h. Afterwards, the cells were fixed in 4% paraformaldehyde in PBS (pH 7.4) for 15 min, permeabilized with 0.1% Triton X-100 for another 15 min and blocked with Ultracruz Blocking reagent (sc-516214, Santa Cruz, CA, USA) for 1 h. At the end of this time, they were incubated with monoclonal antibody Mdr-1 conjugated to Alexa Fluor 488 (sc-55510 AF488, 1:200) at 4 °C overnight. Subsequently, the cells were washed three times, and DAPI (ENZ-53003) reagent was used to counterstain the nuclei. Finally, immunofluorescence images were examined through an inverted fluorescence microscope (Olympus XI51).

### 4.6. Assessment of the Intracellular Accumulation of Doxorubicin

The intracellular accumulation of doxorubicin, a P-gp substrate, was determined in K562 and K562-RI cells to appraise P-gp activity. Briefly, the cells (1 × 10^6^ cells) were seeded in triplicate in 25 cm^2^ plates and exposed to doxorubicin (10 μg/mL) in the presence or absence of ketoconazole (10 μM) for 1 h. Control cells were only in contact with the vehicle. The cells were centrifuged after incubation, and the pellets were washed three times with ice-cold phosphate buffer solution (PBS). Subsequently, the cells were analyzed by flow cytometry (Guava^®^ easyCyte, Merck Millipore), obtaining data from 10,000 acquired events with InCyte software (Merck Millipore, Darmstadt, DE, USA). The fluorescence of doxorubicin was quantified at 488 nm excitation and 575 nm emission wavelength.

### 4.7. Determination of Apoptosis by Flow Cytometry

Externalization of phosphatidylserine was evaluated with the Annexin-V-FLUOS Staining Kit. Cells (1 × 10^6^) were seeded in Costar^®^ 6-well Clear TC-treated Plates and exposed with imatinib alone (200 nM), ketoconazole alone (10 μM), or imatinib plus ketoconazole for 96 h. At the end of the incubation period, the cells were harvested, centrifuged at 2000 rpm for 5 min, washed once with PBS, and centrifuged again. Then they were resuspended in Annexin-V-FLUOS labeling solution, Annexin V-FITC (FITC), and propidium iodide (PI), to be incubated for 15 min at room temperature in the dark, according to the Annexin-V-FLUOS Staining Kit protocol. Flow cytometry was carried out to obtain 5 × 10^3^ cells. The analysis of annexin was conducted with the BD FACS Canto II BD flow cytometry system, and BD FACSDiva software 6.0. The results were expressed as the total percentage of cells undergoing apoptosis. At least three independent experiments were performed for each assay.

## 5. Conclusions

The current study provides evidence that P-gp, a drug efflux pump, plays an important role in the development of resistance to imatinib in CML cells. In vitro testing presently showed the capacity of an inhibitor of P-gp, ketoconazole, to reverse CML cell resistance to imatinib treatment. In resistant CML cells, the antifungal ketoconazole inhibited the overexpression and efflux function of P-gp. Additionally, it increased the intracellular concentration of doxorubicin (a P-gp substrate) when resistant cells were incubated with doxorubicin plus ketoconazole. Thus, a possible corresponding increment in the intracellular concentration of imatinib may have taken place when resistant cells were incubated with this drug plus ketoconazole. Finally, ketoconazole triggered greater apoptosis of resistant CML cells. According to these findings on the mechanisms of action of ketoconazole in CML cells resistant to imatinib, the administration of imatinib plus ketoconazole is likely to favor reversal of resistance in CML patients treated unsuccessfully with imatinib alone; therefore, we propose the repositioning of ketoconazole for the treatment of CML in patients resistant to imatinib.

## Data Availability

The data that support the findings of this study are available on request to the corresponding author.
